# Curcumin restores sensitivity to retinoic acid in triple negative breast cancer cells

**DOI:** 10.1186/1471-2407-14-724

**Published:** 2014-09-27

**Authors:** Padmamalini Thulasiraman, Daniel J McAndrews, Imran Q Mohiudddin

**Affiliations:** Department of Biomedical Sciences, College of Allied Health, University of South Alabama, Mobile, Al, USA

**Keywords:** Curcumin, Retinoic acid, Triple negative breast cancer, Fatty acid binding protein 5, Peroxisome proliferator-activated receptor β/δ

## Abstract

**Background:**

A major obstacle in the use of retinoid therapy in cancer is the resistance to this agent in tumors. Retinoic acid facilitates the growth of mammary carcinoma cells which express high levels of fatty acid-binding protein 5 (FABP5). This protein delivers retinoic acid to peroxisome proliferator-activated receptor β/δ (PPARβ/δ) that targets genes involved in cell proliferation and survival. One approach to overcome resistance of mammary carcinoma cells to retinoic acid is to target and suppress the FABP5/ PPARβ/δ pathway. The objective of this research was to investigate the effect of curcumin, a polyphenol extract from the plant Curcuma longa, on the FABP5/ PPARβ/δ pathway in retinoic acid resistant triple negative breast cancer cells.

**Methods:**

Cell viability and proliferation of triple negative breast cancer cell lines (MDA-MB-231 and MD-MB-468) treated with curcumin and/or retinoic was analyzed using 3-(4,5-dimethylthiazol-2-yl)-2,5-diphenyltetrazolium bromide (MTT) and 5-bromo-2’-deoxyuridine (BrdU). Expression level of FABP5 and PPARβ/δ in these cells treated with curcumin was examined by Western Blotting analysis and Quantitative Real-Time Polymerase Chain Reaction (qRT-PCR). Effect of curcumin and retinoic acid on PPARβ/δ target genes, PDK1and VEGF-A were also examined using qRT-PCR. Western Blotting was utilized to examine the protein expression level of the p65 subunit of NF-κB.

**Results:**

Treatment of retinoic acid resistant triple negative breast cancer cells with curcumin sensitized these cells to retinoic acid mediated growth suppression, as well as suppressed incorporation of BrdU. Further studies demonstrated that curcumin showed a marked reduction in the expression level of FABP5 and PPARβ/δ. We provide evidence that curcumin suppresses p65, a transcription factor known to regulate FABP5. The combination of curcumin with retinoic acid suppressed PPARβ/δ target genes, VEGF-A and PDK1.

**Conclusions:**

Curcumin suppresses the expression level of FABP5 and PPARβ/δ in triple negative mammary carcinoma cells. By targeting the FABP5/PPARβ/δ pathway, curcumin prevents the delivery of retinoic acid to PPARβ/δ and suppresses retinoic acid-induced PPARβ/δ target gene, VEGF-A. Our data demonstrates that suppression of the FABP5/ PPARβ/δ pathway by curcumin sensitizes retinoic acid resistant triple negative breast cancer cells to retinoic acid mediated growth suppression.

## Background

Although breast cancer is the second most leading cause of death among women in the western world, early detection and new treatments have improved survival rates [1]. However, no effective treatment for metastatic, triple negative breast cancer (TNBC) is available following surgery, radiation and chemotherapy for the primary tumor [2, 3]. This subtype of breast cancer accounts for 15-20% of all breast cancers and the signature of TNBCs is the lack of the estrogen receptor, progesterone receptor and the lack of the overexpression of HER2 [4, 5]. Drug resistance is a major problem in TNBC patients, promoting the need to understand the molecular mechanisms involved in the disease and identify future targeted therapy. Several promising agents are currently under clinical trials for the prevention of TNBC which include poly (ADP-ribose) polymerase inhibitors, vitamin D and rexinoids [6].

Anticancer drugs, derived from natural sources, have been used alone or in combination with traditional drugs to treat multiple diseases, including cancer [7–9]. Curcumin derived from the plant Curcuma longa has been used as a dietary agent, food preservative and a longtime favorite Asian medicinal treatment [10]. It is a hydrophobic polyphenol derived from turmeric (*Curcuma longa)* that has anti-oxidant, anti-inflammatory and anti-cancer properties, promoting its potential for targeting various diseases, including cancer, arthritis, atherosclerosis, diabetes, and auto-immune diseases [11, 12]. Curcumin has exhibited inhibitory effects on several malignant cancers, including breast cancer [13–16]. It has been used in clinical trials for colorectal cancer [17] and pancreatic cancer [18], and its use in combination with other therapeutic drugs promotes the suppression of tumor growth [19–21]. Due to the low bioavailability and high metabolic instability of curcumin, development of analogs of curcumin and nanocurcumin to improve their chemotherapeutic efficacies are being investigated as next generation targeted therapy [22, 23]. Despite its current limitations, curcumin is highlighted for its efficacy in chemoprevention and reversing chemo-resistance in certain tumors [24–26]. The ability of curcumin and its analogs to enhance the efficacy of existing chemotherapeutic agents will add value for its use in the treatment of highly aggressive chemo-resistant breast tumors.

The effect of curcumin is in part due to its ability to interfere with multiple signaling cascades such as cell cycle regulators, apoptotic proteins, pro-inflammatory cytokines, proliferative regulators and transcription factors such as nuclear factor-kappa B (NF-κB) and Stat3 [27]. It inhibits cancer cell and tumor growth, suppresses proliferation, and blocks angiogenesis and inflammation. Due to its pleiotropic effect, the role of curcumin to regulate various signaling pathways and genes have been reported in different cancer cell lines [28].

The use of retinoid therapy in cancer is promoted by the ability of retinoids to induce differentiation, cell cycle cycle arrest and apoptosis [29, 30]. Due to its favorable effect on the treatment of acute promyelocytic leukemia, retinoids are being tested in clinical trials in several tumor types [31]. Vitamin A metabolite, retinoic acid (RA) transduces its signals by binding to specific nuclear hormone receptors termed retinoic acid receptors (RAR), which include RAR α, β, and γ [32]. These receptors exist as predominately RAR/RXR heterodimers and to a lesser extent RXR/RXR homodimer [33, 34]. RARs bind to all-*trans*-RA (ATRA) or 9-*cis*-retinoic acid, whereas RXRs bind specifically to 9-*cis*-retinoic acid. The ligand-receptor complex acts as a transcription factor which binds to a specific DNA sequence element found on the promoter regions of target genes called retinoic acid response element (RARE) [32–35]. Transcriptional activation of RARs leads to differentiation and growth arrest [36, 37], as well as apoptosis [38–41], establishing a prominent role of its use in anti-cancer therapy [31]. Interestingly, RA has an alternative function and in some tissues RA promotes cell growth [41–46], and paradoxically facilitates tumor development. It has been well established that RA translates its pro-carcinogenic properties in a RAR-independent mechanism through activation of the nuclear receptor, peroxisome proliferator-activated receptor β/δ (PPARβ/δ) and its target genes [41, 42]. Studies have shown that fatty-acid binding protein 5 (FABP5) facilitates the transfer of ligands from the cytoplasm to PPARβ/δ, which enhances PPARβ/δ target genes that are directly involved in proliferative responses and cell survival, promoting cell growth and protection against apoptosis [47, 48]. PPARβ/δ has been implicated in the growth of other human cancers, including lung carcinoma, breast cancer and colon cancer [49]. This nuclear receptor is well known to regulate the expression of angiogenic factor, vascular endothelial growth factor A (VEGF-A), pro-survival signals of PDK1/Akt, and anti-apoptotic protein, 14-3-3epsilon [41, 42, 50].

The importance of FABP5 as a prognostic marker in breast cancer patients was studied in a cohort of breast tissues, pinpointing that elevated levels of FABP5 was correlated with tumor grade and poor prognosis [51]. Not only are elevated levels of FABP5 a key determinant in the tumorigenic properties of mammary carcinoma [52], but also pancreatic cancer cell subtypes with elevated levels of FABP5 were associated with migration and invasion of cells, paralleling to lack of tumor growth inhibition [53]. High FABP5 protein expression was evident in short term glioblastoma survivors with highly proliferating tumors compared to long term glioblastoma survivors [54]. In light of the mounting evidence on the role of FABP5 in cancer cell lines, FABP5 may serve as a novel prognostic marker and inhibiting FABP5 can serve as a potential combinatorial treatment to sensitize mammary carcinoma cells to retinoid therapy.

In this study, we report that curcumin blocks cell proliferation in RA-resistant TNBC cell lines by inhibiting FABP5. It sensitizes these cells to RA mediated growth suppression. The effect of curcumin in enhancing the inhibitory effects of RA in TNBC cells is the result of diminished expression of FABP5 and PPARβ/δ. Furthermore, we have identified a possible mechanism responsible for growth suppression by curcumin and RA. Understanding the mechanisms by which curcumin reverses the resistance of breast cancer to RA may provide alternate treatments for RA-resistant TNBC patients.

## Methods

### Reagents

As previously described [55, 56], antibodies against FABP5 were obtained from R & D systems and β-tubulin was purchased from Sigma Aldrich Co (St. Louis, MO). Antibodies for PPARβ/δ and p65 were obtained from Santa Cruz (Santa Cruz, CA) and Cell Signaling (Boston, MA), respectively. Use of PPARβ/δ and p65 antibodies was referenced in [57, 58], respectively. Antibody against β-actin was purchased from Cell Signaling (Boston, MA). Anti-mouse and anti-rabbit immunoglobulin horseradish peroxidase-conjugated antibodies were from BioRad, and anti-goat immunoglobulin was from Santa Cruz. Curcumin (C-1386) and all-*trans*-retinoic acid (R-2625) were obtained from Sigma. MTT reagent (3-(4, 5-dimethylthiazol-2-yl)-2,5-diphenyl tetrazolium bromide) was purchased from Sigma.

### Cells

MDA-MB-231, MDA-MB-468, SkBr3 and MCF-7 cells were maintained in Dulbecco’s Modified Eagle’s Medium (DMEM) supplemented with 10% fetal bovine serum (FBS) and antibiotics. MDA-MB-468 was purchased from American Type Culture Collection (Manassas, VA).

### RA and Curcumin Preparation

A small amount of all-*trans-*RA was added to a 1 mL aliquot of 100% ethanol in the dark and rotated for 45 minutes at 4°C. The absorbance of RA was measured at 350 nm and the extinction coefficient of RA (ϵ = 45,300 M^-1^ cm^-1^) was used to calculate the concentration of RA. A Stock of 1 mM was prepared and used for treatment of cells. Curcumin was freshly prepared each time at a stock concentration of 50 mM in dimethyl sulfoxide (DMSO) and diluted at the appropriate concentration for treatment of cells.

### Western blots

Cells were cultured in 100 mm plates and treated with DMSO or curcumin for 24 hours. Cells were lysed in a buffer containing 150 mM NaCl, 10 mM Tris, pH 7.2, 0.1% SDS, 1% Triton X-100, 1% deoxycholate, 5 mM EDTA, and 1 mM PMSF. Cells were lysed on ice for 1 hour and protein concentration was determined by the Bradford Assay. Cell lysate was resolved by SDS-PAGE and probed using the appropriate antibody. Anti-β-tubulin or anti-β-actin was used for loading control.

### Quantitative Real-Time Polymerase Chain Reaction (qRT-PCR)

Cells were treated with curcumin and/or ATRA, and RNA was extracted using Trizol (Life Technologies, Grand, Island, NY). As described in the high capacity RNA to cDNA kit from Applied Biosystems (Gaitherburg, MD), 2 μg total RNA was reverse transcribed into cDNA. To determine expression of FABP5, PPARβ/δ and PDK1, and VEGF-A, qRT-PCR was carried out by using commercially available Taqman Chemistry and Assay on Demand Probes (Applied Biosystems). GAPDH was used for normalization. Detection and data analysis were carried out on the ABI Step One Plus Real-Time PCR System.

### siRNA knockdown experiments

NF-κB p65 siRNA was purchased from Santa Cruz Biotechnology. Briefly, 2 × 10 ^5^ MDA-MB-231 cells were plated in 6-well plates and transfected with 1 μg of p65 siRNA or control siRNA oligos using lipofectamine 2000 as per the manufacturer’s instructions. Cells were incubated for 24 hours after which cells were harvested and analyzed for p65 and FABP5 expression using Western blot analysis.

### Cell Proliferation MTT Assay

MDA-MB-231 and MDA-MB-468 cells (2500 cells/well) were seeded in a 96 well plate with Dulbecco’s modified Eagle’s medium, 10% charcoal treated FBS and supplemented with antibiotics, and allowed to adhere overnight. Cells were then treated with 30 uM curcumin and/or 1 uM ATRA for 48 hours. Controls were treated with 0.1% DMSO, 0.1% ethanol and/or the combination of both. After 48 hours, 5 μg/ml MTT reagent (3-(4, 5-dimethylthiazol-2-yl)-2, 5-diphenyl tetrazolium bromide) was added directly to the cells for 3 hours, or until crystals formed. The media was carefully removed from the plate, leaving the cells intact and the cells were then resuspended in 150 μl of 0.04 M HCl in isopropanol. Absorbance was read at 570 nm to determine cell proliferation.

### BrdU (5-bromo-2’-deoxyuridine) cell proliferation assay

Cell Proliferation was also measured by the incorporation of BrdU in cells by using the BrdU cell proliferation kit from Cell Signaling. MDA-MB-231 cells were plated in 96-well plates and treated with 30 uM curcumin and/or 1 uM ATRA for 48 hours. Controls were treated with 0.1% DMSO, 0.1% ethanol and/or the combination of both. Cells were then pulsed with BrdU overnight, fixed and followed by immunodetection of the incorporation of BrdU label. Absorbance was read at 450 nm to determine cell proliferation.

### Statistical analysis

Statistical significance of differences between treatments was determined using two tailed student *t*-test and p values were noted. Differences between groups were considered statistically significant at p < 0.05.

## Results

### Curcumin and ATRA suppress growth of RA-resistant TNBC cell lines

**Figure 1:**
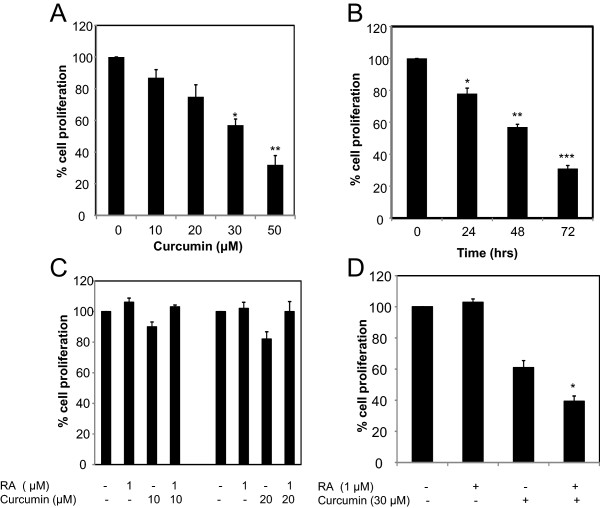
**Curcumin sensitizes MDA-MB-231 cells to RA-mediated growth suppression. (A)** MDA-MD-231 cells were treated with varying dose of curcumin for 48 hours. Control was DMSO. The % cell proliferation for each of the treatment with curcumin for the designated concentration was calculated with respect to the treatment with DMSO. Data are mean of ± SE (n = 3). *p = 0.008 versus control, **p = 0.007 versus control **(B)** MDA-MD-231 cells were treated with 30 μM curcumin for varying time. Control was DMSO. The % cell proliferation for each of the treatment with curcumin for the designated time was calculated with respect to the treatment with DMSO for the corresponding time. Data are mean of ± SE (n = 3). *p = 0.0025 versus control, **p = 0.002 versus control, ***p = 0.0007 versus control. **(C)** MDA-MB-231 cells were treated with 1 μM ATRA (RA), 10 μM or 20 μM curcumin and the combination of both agents for 48 hours. Control was DMSO, ethanol or a combination of both. The % cell proliferation for each of the treatment (curcumin, ATRA or both) was calculated relative to their solvent, DMSO, ethanol or DMSO and ethanol, respectively. The controls were set at 100%. Data are mean of ± SE (n = 3). **(D)** MDA-MB-231 cells were treated with 1 μM ATRA, 30 μM curcumin, or 30 μM curcumin and 1 μM ATRA for 48 hours. Control was DMSO, ethanol or a combination of both. The % cell proliferation for each of the treatment (curcumin, ATRA or both) was calculated relative to their solvent, DMSO, ethanol or DMSO and ethanol, respectively. The controls were set at 100%. Data are mean of ± SE (n = 4). *p = 0.000036 versus curcumin treatment.

To examine the effect of curcumin and ATRA on cell viability, MTT assay was performed in two RA-resistant TNBC cell lines. Mammary carcinoma cell, MDA-MB-231 is sensitive to curcumin [59] and resistant to ATRA [60]. As shown in Figure 1A, curcumin suppresses mammary carcinoma cell growth of MDA-MB-231 cells in a dose dependent manner (Figure 1A). By 48 hours, 30 μM curcumin alone had produced a growth inhibitory effect of approximately 40% in MDA-MB-231 cells. As illustrated in Figure 1B, the response to 30 μM of curcumin to suppress cell proliferation of MDA-MB-231 cells is also time dependent (Figure 1B). Thus, the proliferation of MDA-MB-231 cells was inhibited by curcumin in a dose and time-dependent manner. To assess whether curcumin could sensitize these cells to RA-mediated growth suppression, we performed MTT assay in MDA-MB-231 cells treated with varying doses of curcumin in the presence or absence of 1 μM ATRA. As expected, MDA-MB-231 cells treated only with ATRA were resistant to cell growth suppression while 10 μM or 20 μM curcumin suppressed growth, although not statistically significant (Figure 1C). The combination of ATRA with 10 μM or 20 μM of curcumin did not effect cell growth (Figure 1C). In fact, cell growth of MDA-MB-231 cells treated with curcumin and ATRA was similar to ATRA-treated cells, suggesting that the proliferative function of RA suppresses the anti-proliferative effects of curcumin. As expected, 30 μM curcumin suppressed mammary carcinoma cell growth by 40% (Figure 1d) and interestingly, MDA-MB-231 cells treated with a combination of 30 μM curcumin and 1 μM ATRA further reduced cell growth to approximately 60%, sensitizing TNBC MDA-MB-231 cells to the inhibitory effects of RA (Figure 1d). At a maximal dose of 30 μM curcumin, this agent was able to sensitize MDA-MB-231 cells to RA.

**Figure 2:**
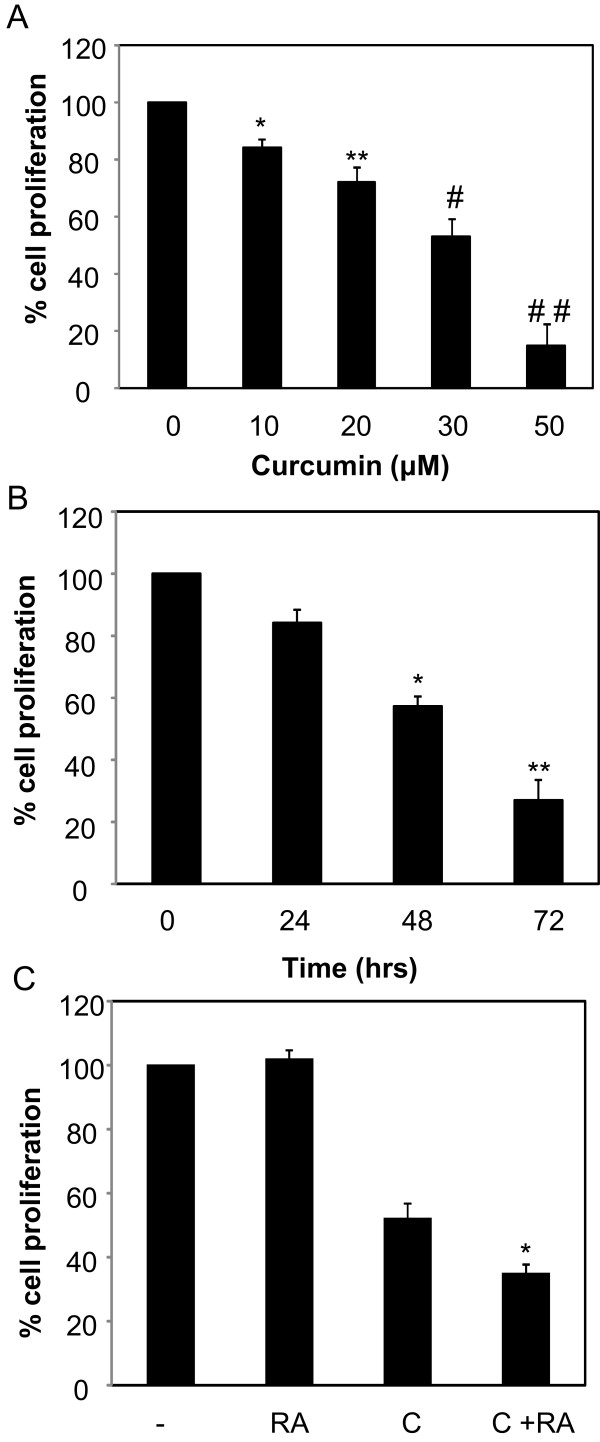
**RA-resistant MDA-MB-468 cells are sensitized to ATRA by curcumin. (A)** MDA-MD-468 cells were plated in 96 well plates, treated with varying dose of curcumin for 48 hours and the control used was DMSO. The % cell proliferation for each of the treatment with curcumin for the designated concentration was calculated with respect to the treatment with DMSO for the corresponding dose. Data are mean of ± SE (n = 3). * p = 0.04 versus control, ** p = 0.04 versus control, # p = 0.02 versus control, # # p = 0.008. **(B)** MDA-MD-468 cells were plated in 96 well plates, treated with 30 μM curcumin for varying time. The control was DMSO treated according to the timeframe of the cells treated with curcumin. The % cell proliferation for each of the treatment with curcumin for the designated time was calculated with respect to the treatment with DMSO for the corresponding time. Data are mean of ± SE (n = 3). * p = 0.009 versus control, ** p = 0.009 versus control. **(C)** MDA-MB-468 cells (2500 cells/well) were plated in 96 well plates, treated with 1 μM ATRA (RA), 30 μM curcumin (C), or 30 μM curcumin and 1 μM ATRA for 48 hours. The control was DMSO, ethanol or a combination of both. The % cell proliferation for each of the treatment (curcumin, ATRA or both) was calculated relative to their solvent, DMSO, ethanol or DMSO and ethanol, respectively. The controls were set at 100%. Data are mean of ± SE (n = 4). * p = 0.03 versus curcumin.

To test whether sensitivity to ATRA can be attained by treatment of another RA-resistant breast cancer cells with curcumin, we performed cell proliferation assay in TNBC MDA-MB-468 cells. The growth inhibitory effect of curcumin was observed in MDA-MB-468 cells (Figure 2a) and 30 μM curcumin suppressed the proliferation of these cells in a time dependent manner (Figure 2b). Similar to the inhibitory effects of 30 μM curcumin and 1 μM ATRA in MDA-MB-231 cells (Figure 1d), we also observed similar results in MDA-MB-468. Curcumin reduced growth of MDA-MB-468, while no such inhibition was observed in the presence of ATRA (Figure 2c). However, the combination of both 1 μM ATRA and 30 μM curcumin reduced growth of MDA-MB-468 when compared to either treatment alone (Figure 2c). Thus, curcumin sensitizes RA-resistant TNBC cells to the growth suppressive effects of RA.

### Curcumin down-regulates FABP5 and PPARβ/δ expression level in MDA-MB-231 and MDA-MB-468

**Figure 3:**
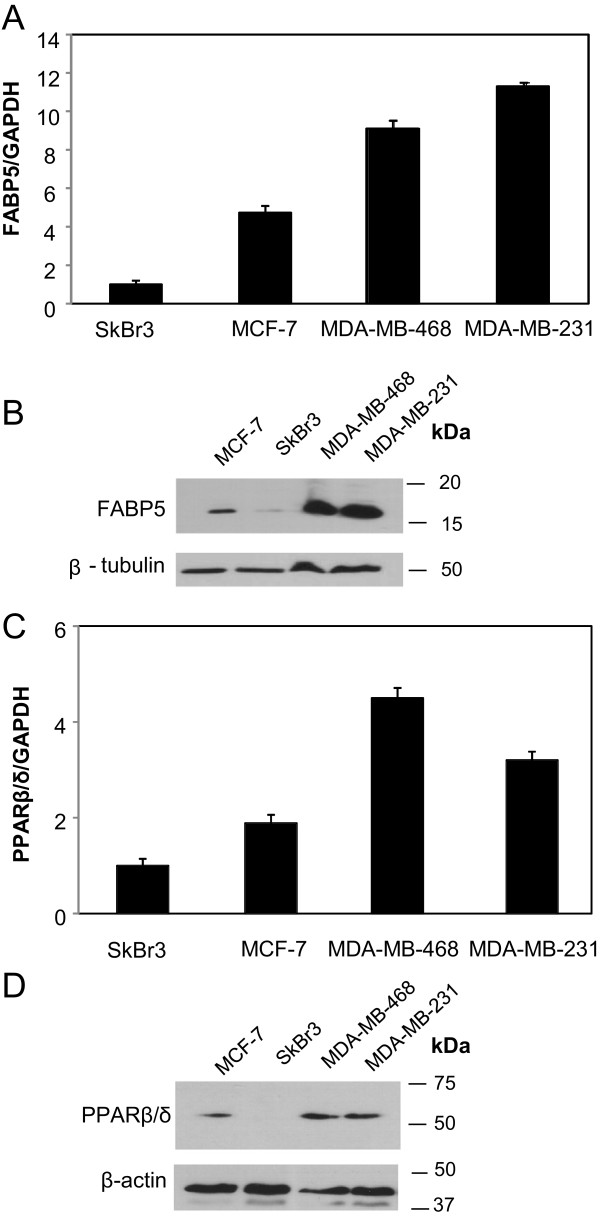
**Expression levels of FABP5 and PPAR** **β/δ** **in breast cancer cell lines. (A)** Basal expression level of FABP5 was analyzed by qRT-PCR in several breast cancer cell lines. GAPDH was used for normalization. Data are mean of ± SE (n = 3). **(B)** Basal expression level of FABP5 was analyzed by immunoblotting in several breast cancer cell lines. β-tubulin was used as a loading control **(C)** Basal expression level of PPARβ/δ was analyzed by qRT-PCR in several breast cancer cell lines. GAPDH was used for normalization. Data are mean of ± SE (n = 3). **(D)** Basal expression level of PPARβ/δ was analyzed by immunoblotting in several breast cancer cell lines. β-actin was used as a loading control.

Sensitivity to RA is dependent on the expression level of FABP5 in mammary carcinoma cells [41, 42, 51, 52]. We examined the expression level of FABP5 in RA-resistant TNBC cell lines (MDA-MB-231 and MDA-MB-468) in comparison to mammary carcinoma cells sensitive to RA-mediated growth suppression. High mRNA expression level of FABP5 was evident in the highly aggressive, RA-resistant TNBC cell lines (Figure 3a), MDA-MB-231 and MDA-MB-468, which are both ER- and HER2- in comparison to MCF-7 (ER+/HER2-) and SkBr3 (ER-/HER2+). Similarly, there is high FABP5 protein expression in MDA-MB-231 and MDA-MB-468 cell lines compared to MCF-7 and SkBr3 cells (Figure 3b). Although SkBr3 mammary carcinoma cells are aggressive and HER2+, these cells are sensitive to retinoic acid [61, 62], as is MCF-7 cells. It has been well established that FABP5 suppresses sensitivity of cancer cells to retinoic acid [41, 51], and the fact that there is low expression level of FABP5 in SkBr3 suggests sensitivity of these cells to retinoic acid. Thus, a direct relationship exists between the high expression level of FABP5 and the lack of sensitivity to retinoic acid, and not the aggressiveness of the cancer.

**Figure 4:**
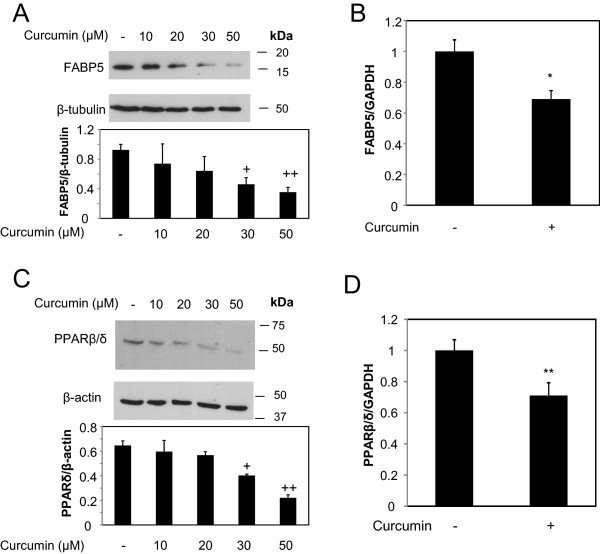
**Curcumin suppresses the expression level of FABP5 and PPARβ** **/δ** **in MDA-MB-231 cells. (A)** MDA-MB-231 cells were treated with varying concentration of curcumin for 24 hours (0.1% DMSO was used for the control) prior to cell lysis. Cell lysates were resolved with SDS-PAGE and immunoblotted with antibodies recognizing FABP5, and β-tubulin was used as a loading control (top panel). Bottom panel: Densitometry analyses provide the relative amount of FABP5 normalized to β-tubulin. The analysis was performed in triplicates as mean of ± SE. +, p = 0.04 versus control, + +, p = 0.03 versus control. **(B)** Total RNA was collected from MDA-MB-231 cells treated with curcumin (30 μM) for 4 hours or 0.1% DMSO as control. Expression level of FABP5 was analyzed by qRT-PCR. GAPDH was used for normalization. Data are mean of ± SE (n = 3). * p = 0.0002 versus control treatment **(C)** MDA-MB-231 cells were treated with varying concentration of curcumin for 24 hours (0.1% DMSO was used for the control) prior to cell lysis. Cell lysates were resolved with SDS-PAGE and immunoblotted with antibodies recognizing PPARβ/δ, and β-actin was used as a loading control (top panel). Bottom panel: Densitometry analyses provide the relative amount of PPARβ/δ normalized to β-actin. The analysis was performed in triplicates as mean of ± SE. +, p = 0.04 versus control, + +, p = 0.02 versus control **(D)** Total RNA was collected from MDA-MB-231 cells treated with curcumin (30 μM) for 4 hours or 0.1% DMSO as control. Expression level of PPARβ/δ was analyzed by qRT-PCR. GAPDH was used for normalization. Data are mean of ± SE (n = 3)** p = 0.0003 versus control treatment.

**Figure 5:**
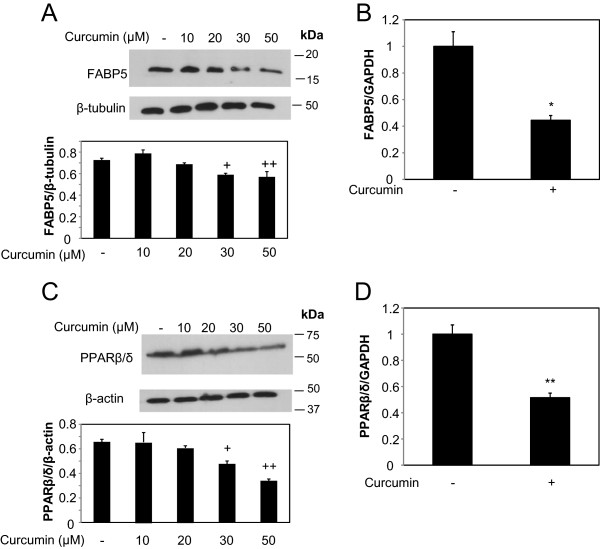
**Curcumin suppresses the expression level of FABP5 and PPARβ** **/δ** **in MDA-MB-468 cells. (A)** MDA-MB-468 cells were treated with 30 μM of curcumin for 24 hours (0.1% DMSO was used for the control) prior to cell lysis. Cell lysates were resolved with SDS-PAGE and immunoblotted with antibodies recognizing FABP5, and β-tubulin was used as a loading control. Bottom panel: Densitometry analyses provide the relative amount of FABP5 normalized to β-tubulin. The analysis was performed in triplicates as mean of ± SE. +, p = 0.02 versus control, + +, p = 0.04 versus control **(B)** Total RNA was collected from MDA-MB-468 cells treated with curcumin (30 μM) for 4 hours or 0.1% DMSO as control. Expression level of FABP5 was analyzed by qRT-PCR. GAPDH was used for normalization. Data are mean of ± SE (n = 3). * p = 0.03 versus control treatment. **(C)** MDA-MB-468 cells were treated with varying concentration of curcumin for 24 hours (0.1% DMSO was used for the control) prior to cell lysis. Cell lysates were resolved with SDS-PAGE and immunoblotted with antibodies recognizing PPARβ/δ, and β-actin was used as a loading control (top panel). Bottom panel: Densitometry analyses provide the relative amount of PPARβ/δ normalized to β-actin. The analysis was performed in triplicates as mean of ± SE. +, p = 0.04 versus control, + +, p = 0.007 versus control. **(D)** Total RNA was collected from MDA-MB-468 cells treated with curcumin (30 μM) for 4 hours or 0.1% DMSO as control. Expression level of PPARβ/δ was analyzed by qRT-PCR. GAPDH was used for normalization. Data are mean of ± SE (n = 3)** p = 0.03 versus control treatment.

The mRNA and protein expression of the nuclear receptor, PPARβ/δ which binds to ligands delivered by FABP5 was also examined in the various breast cancer cell lines. High mRNA and protein expression level of PPARβ/δ was evident in the RA-resistant mammary carcinoma cells, MDA-MB-231 and MDA-MB-468 (Figure 3c and d) when compared to MCF-7 cells. However, the highly aggressive SkBr3 had low levels of PPARβ/δ mRNA expression and undetectable levels of PPARβ/δ protein expression (Figure 3c and d). This suggests there is no correlation between the level of PPARβ/δ and the aggressiveness of the breast cancer cells, but instead an association between RA-resistance and high expression of PPARβ/δ.

As shown in Figure 1d, 30 μM of curcumin suppressed RA- resistant MDA-MB-231 cells, while the combination with 1 μM RA sensitized these cells to respond to retinoic acid-mediated growth suppression. To determine whether the enhanced growth-inhibitory effects of curcumin and RA on mammary carcinoma cells were likely due to associated changes of FABP5 expression, we sought to assess the effect of curcumin on FABP5 expression in MDA-MB-231 cells. To investigate whether curcumin regulates the expression level of FABP5, MDA-MB-231 cells were treated with varying concentrations of curcumin for 24 hours, and the levels of FABP5 protein were determined by Western blot analysis. Protein levels of FABP5 were reduced dose-dependently in MDA-MB-231 cells after treatment with curcumin (Figure 4a). A significant decrease in FABP5 protein expression was evident at 30 μM curcumin (Figure 4a). The levels of FABP5 at mRNA level were also analyzed by qRT-PCR. To analyze the mRNA expression level of FABP5, MDA-MB-231 cells were treated with 30 μM curcumin, mRNA isolated and consistent with reduced protein expression (Figure 4a), 30 μM curcumin suppressed mRNA expression levels of FABP5 by approximately 30% (Figure 4b). At 30 μM curcumin, FABP5 expression was reduced in MDA-MB-231 cells (Figure 4a, b), and at the same dose, curcumin sensitized them to RA-mediated growth suppression (Figure 1d).

**Figure 6:**
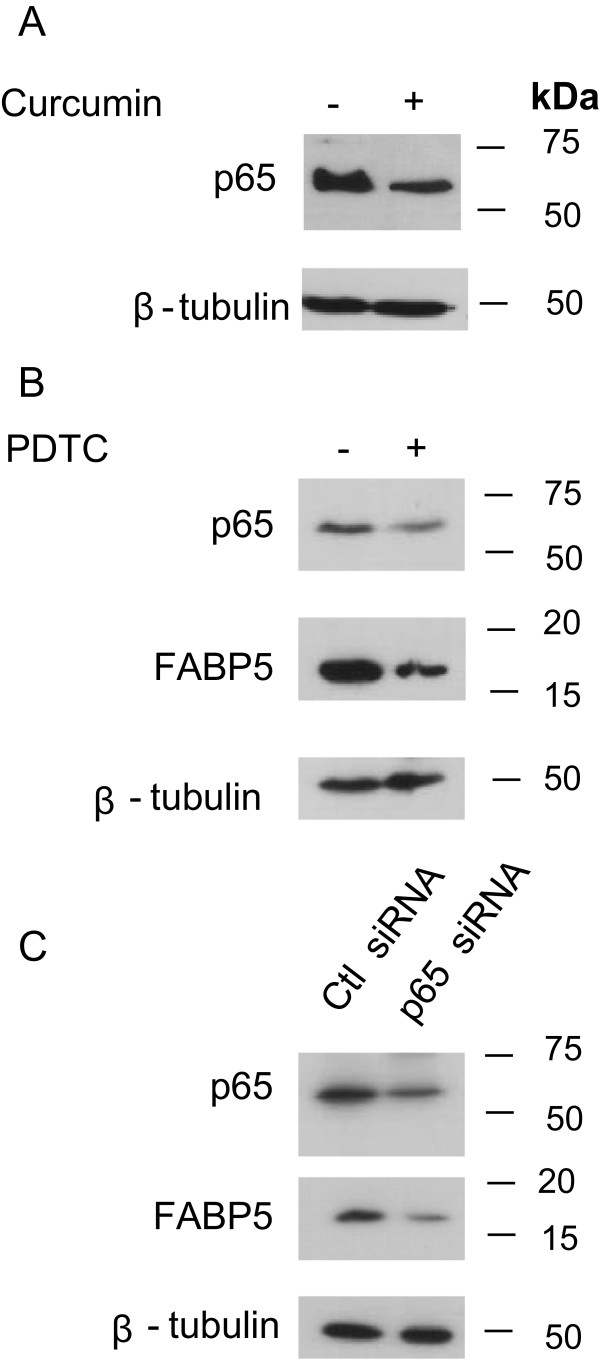
**Curcumin regulates FABP5 via NF-κ** **B. (A)** MDA-MB-231 cells were treated with 30 μM curcumin, total protein extracted and probed for p65. β-tubulin was used as a loading control **(B)** MDA-MB-231 cells were treated with 25 μM PDTC, cell lysates were resolved with SDS-PAGE and immunoblotted with antibodies recognizing p65 or FABP5. β-tubulin was used as a loading control. **(C)** MDA-MB-231 cells were transfected with control and p65 siRNA for 24 hours, protein extracted and expression level of p65 and FABP5 was examined by immunoblotting with antibodies recognizing p65 and FABP5, respectively. β-tubulin was used as a loading control.

**Figure 7:**
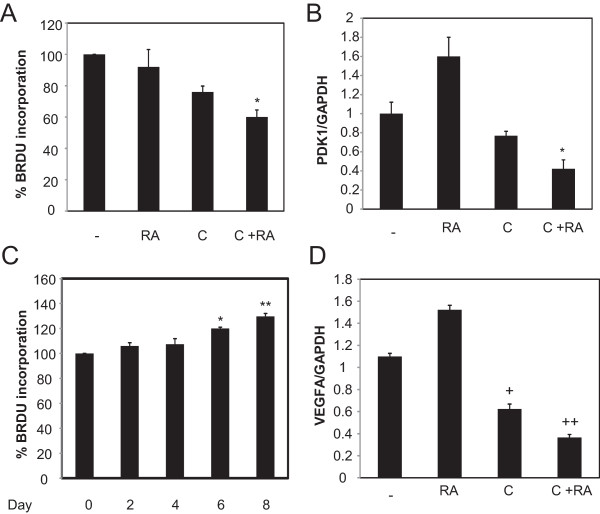
**Curcumin and ATRA reduce VEGF-A expression in MDA-MB-231 cells. (A)** MDA-MB-231 cells were plated in 96 well plates treated with 1 μM ATRA (RA), 30 μM curcumin (C) or 30 μM curcumin and 1 μM ATRA for 48 hours. The control was DMSO, ethanol or a combination of both. The % BrdU incorporation for each of the treatment (curcumin, ATRA or both) was calculated relative to their solvent, DMSO, ethanol or DMSO and ethanol, respectively. Data are mean of ± SE (n = 3). *, p = 0.035 versus curcumin. **(B)** MDA-MB-231 cells were treated with 30 μM curcumin (C) for 24 hours prior to the addition of 1 μM ATRA (RA) for 4 hours. Total RNA was collected from MDA-MB-231, qRT-PCR was performed and expression levels of PDK1 were measured. GAPDH was used for normalization. Data are mean of ± SE (n = 3). *, p = 0.012 versus curcumin. **(C)** MDA-MB-231 cells were plated in 96 well plates treated with 1 μM ATRA for 2–8 days. The % BrdU incorporation for each of the treatment was calculated relative to their solvent, DMSO. Data are mean of ± SE (n = 3). *, p = 0.0008 versus control ** p = 0.002 versus control. **(D)** MDA-MB-231 cells were treated with 30 μM curcumin (C) for 24 hours prior to the addition of 1 μM ATRA (RA) for 4 hours. Total RNA was collected from MDA-MB-231, qRT-PCR was performed and expression levels of VEGF-A was measured. GAPDH was used for normalization. Data are mean of ± SE (n = 3). +, p = 0.016 versus control, ++, p = 0.03 versus curcumin.

It is known that FABP5 delivers ligands, such as RA, to the nuclear receptor PPARβ/δ [47, 48], known to target genes involved in cell survival and proliferation. Since curcumin downregulates the expression of FABP5 (Figure 4a and b), we investigated the effect of curcumin on PPARβ/δ. MDA-MB-231 cells were treated with varying doses of curcumin and at 30 μM of curcumin, a statistically significant decrease in PPARβ/δ protein expression level was observed (Figure 4c), concomitant to the decrease of FABP5 expression by curcumin (Figure 4a and b). Suppression of PPARβ/δ protein expression by curcumin was dose-dependent (Figure 4c). Since 30 μM curcumin inhibited PPARβ/δ protein expression, we also examined PPARβ/δ mRNA expression at this concentration. Our data shows that 30 μM of curcumin reduced PPARβ/δ mRNA in these cells (Figure 4d). Because curcumin affects the expression level of FABP5 and PPARβ/δ in RA-resistant MDA-MB-231 mammary carcinoma cells, we further examined the effect of curcumin on these genes in RA-resistant MDA-MB-468 cells. Curcumin reduced FABP5 protein expression in MDA-MB-468 cells in a dose dependent manner (Figure 5a), with a statistically significant reduction at 30 μM of curcumin. Consistently, FABP5 mRNA expression was also reduced by 30 μM of curcumin (Figure 5b). To examine the effect of curcumin on PPARβ/δ expression, we performed immunoblotting and qRT-PCR to determine the protein and mRNA expression of PPARβ/δ, respectively. Curcumin suppressed PPARβ/δ protein expression in a dose-dependent manner, with a statistically significant effect at 30 μM of curcumin treatment (Figure 5c). Comparable results at the mRNA level of PPARβ/δ was also observed, wherein curcumin suppressed PPARβ/δ mRNA expression levels in MDA-MB-468 cells (Figure 5d).

### Regulation of FABP5 by curcumin is mediated by NF-κB

One of the established biological effects of curcumin to its chemopreventive activity is the inhibition of the NF-κB pathway [27]. Constitutive NF-κB activity is associated with enhanced proliferation and survival of malignant cells [63]. We sought to investigate whether the effect of curcumin on the expression level of FABP5 depends on NF-κB, in particular since it has been described that this transcription factor regulates FABP5 through the two putative NF-κB response elements on the FABP5 promoter [56]. We assessed whether suppression of NF-κB by curcumin regulates the expression level of FABP5 in MDA-MB-231 cells. Equal amounts of cell lysates from control and 30 μM curcumin-treated MDA-MB-231 cells were probed for the NF-κB subunit, p65 using western blot analysis. As reported previously [64] and as shown in Figure 6a, 30 μM curcumin decreased p65 protein expression in MDA-MB-231 cells. To directly test the role of NF-κB to regulate FABP5 in MDA-MB-231 cells, we used pyrrolidine dithiocarbamate (PDTC), a chemical inhibitor on the activation of NF-κB, to decrease p65 expression [65] and examined the effect of FABP5 protein expression. Treatment of MDA-MB-231 cells with 25 μM PDTC suppressed p65 expression level at a modest level (Figure 6b). Concomitantly, we examined the protein expression level of FABP5 in MDA-MB-231 cells treated with PDTC and observed that suppression of the p65 subunit of NF-κB reduced FABP5 expression in MDA-MB-231 cells (Figure 6b). In addition, p65 was silenced in MDA-MB-231 cells and we examined the protein expression of FABP5 (Figure 6c). Knock-down of p65 suppressed FABP5 expression in these cells (Figure 6c). These results support our hypothesis that treatment with curcumin blocks the expression of NF-κB regulated FABP5 gene expression, and that NF-κB is a regulator in the molecular mechanism of curcumin mediated suppression of FABP5.

### Inhibition of PPARβ/δ target gene by curcumin and ATRA

We assessed the population of cells using BrdU incorporation assay upon treatment with curcumin and/or ATRA in MDA-MB-231 cells. Mammary carcinoma cells were treated with either curcumin, ATRA or the combination of both agents for 48 hours and labeled with 5-bromo-2-deoxyuridine (BrdU) overnight prior to analysis. As expected, treatment of MDA-MB-231 cells with ATRA did not reduce cell proliferation (Figure 7a). However, MDA-MB-231-treated with curcumin showed a reduction in the population of cells, noted by the reduction in BrdU incorporation, and the effect was pronounced when combined with ATRA (Figure 7a). Hence, curcumin and RA together suppress cell proliferation, supporting the results of the MTT assay (Figure 1d).

Activation of PPARβ/δ induces the transcriptional activity of several downstream genes in mammary carcinoma cells such as the survival factor PDK1/Akt, VEGF-A and 14-3-3-epsilon [41, 42, 50]. We examined the effect of curcumin and/or ATRA on the mRNA expression level of PDK1 in MDA-MB-231. As shown in Figure 7b, a 4-hour treatment of MDA-MB-231 cells with 1 μM of ATRA enhanced the mRNA level of the survival factor, PDK1. Although RA enhanced survival factor, PDK1 mRNA expression within 4 hours, enhanced cell proliferation was not observed after 48 hours of treatment with RA and growth of the cells remained as that of the control cells (Figure 7a). The conventional growth inhibitory properties of RA in some cells [35, 37–40, 66] was not observed in triple negative breast cancer cell line, MDA-MB-231 but as noted previously [60] and as seen in Figures 1d and 7a, MDA-MB-231 cells are retinoic acid resistant. However in some carcinomas, RA enhances hyperproliferation [41, 67–69]. To determine whether there is delayed response to hyperproliferation and PDK1 expression, MDA-MB-231were treated with 1 μM ATRA for 2–8 days. At approximately 6 days, RA promoted hyperproliferation of MDA-MB-231 cells (Figure 7c). The timeframe at which PDK1 is induced does not correlate with the time at which MDA-MB-231 cells responds to RA with hyperproliferation, but these results demonstrate that these cells are resistant to the growth inhibitory effects of RA and thus RA-resistant. While ATRA enhanced PDK1 expression, a small reduction (~20%) in mRNA levels was observed when cells were treated with curcumin, though the effect was not statistically significant (Figure 7b). PPARβ/δ ligands have been shown to activate PDK1 expression in several cells [41, 42] but other reports suggest that PDK1 is not a direct target of PPARβ/δ [70, 71]. In our study, we observed that despite the downregulation of PPARβ/δ by curcumin in MDA-MB-231 cells (Figure 4c, d), curcumin does not suppress PDK1 (Figure 7b), suggesting that PDK1 is not regulated by PPARβ/δ in these cells (Figure 7b). However, the combination of curcumin and ATRA further reduced PDK1 at the mRNA level, compared to curcumin alone (Figure 7b). Although PDK1 may not be a PPARβ/δ target gene in these cells, we can conclude that RA regulates PDK1 mRNA expression by an alternative mechanism in these cells. Curcumin may regulate this mechanism and suppress the upregulation of PDK1 mRNA expression by RA.

Due to the controversy that remains on whether PDK1 is a direct target of PPARβ/δ, we also examined the effect of curcumin and RA on another PPARβ/δ target gene, VEGF-A [72]. As shown in Figure 7d, ATRA induced VEGF-A mRNA expression, while curcumin suppressed VEGF-A (Figure 7d). In addition, the combination of curcumin and RA further suppressed VEGF-A expression (Figure 7d). Curcumin-mediated suppression of PPARβ/δ in MDA-MB-231 cells, when compared to control, reduces the downstream target gene, VEGF-A by approximately 40%, while curcumin suppressed RA-mediated transcriptional activity of PPARβ/δ target gene, VEGF-A by an additional 30%. This suggests that the proliferative effect of RA in these cells is diminished in the combination with curcumin, owing to the fact that curcumin suppresses the FABP5/PPARβ/δ pathway, and MDA-MB-231 cells are sensitized to the growth inhibitory effects of RA.

## Discussion

In this study, we show that curcumin sensitizes RA-resistant TNBC cells to RA-mediated growth suppression by modulating the expression level of the FABP5/PPARβ/δ pathway. The high expression level of FABP5 in the aggressive RA-resistant TNBC cells, MDA-MB-231 and MDA-MB-468, correlates with the marked upregulation of FABP5 observed in human breast tumors, particularly in tumors categorized by its invasive properties as well as late stages of breast cancer [51, 52]. Our observation shows that curcumin downregulates FABP5 expression, and concomitantly suppresses its cognate receptor, PPARβ/δ. Adding to the network of genes regulated by curcumin [28], we demonstrate that curcumin regulates the pro-proliferative gene, FABP5 and the nuclear receptor, PPARβ/δ.

Several PPARβ/δ target genes, such as PDK1 and VEGF-A are involved in proliferation and angiogenesis, respectively [41, 42, 72]. In mammary carcinoma cells expressing high levels of FABP5, FABP5 delivers RA to PPARβ/δ, enhances the transcriptional activity of PPARβ/δ and activates PPARβ/δ target gene, PDK1 and VEGF-A [41, 42]. Based on previous reports that RA regulates PDK1 through PPARβ/δ, we also examined the regulation of PDK1 by RA in MDA-MB-231 cells. From our data, we conclude that RA regulates PDK1, however regulation of PDK1 by RA is not via PPARβ/δ in these cells. Several studies have also demonstrated that PDK1 is not a PPARβ/δ target gene [70, 71]. Comparing the suppression of PDK1 by curcumin and/or ATRA suggests that curcumin alone reduces PDK1 expression (not statistically significant), while the combination of the agents further suppress PDK1 mRNA expression. RA may be regulating PDK1, at the mRNA level, by an alternative mechanism. Hence, curcumin may regulate this mechanism and thereby suppress RA-mediated upregulation of PDK1 mRNA. Further studies will be required to dissect the pathway by which RA regulates PDK1 in these cells. Examining the effect of curcumin and ATRA on another PPARβ/δ target gene, VEGF-A, we discovered that RA enhances VEGF-A, while curcumin significantly decreases VEGF-A mRNA expression. Suppression of FABP5 by curcumin reduces the delivery of RA to PPARβ/δ, resulting in diminished transcriptional activation of PPARβ/δ by ATRA. Curcumin suppresses VEGF-A expression by approximately 45%, however in the combination of ATRA, mRNA expression of VEGF-A is reduced to an additional 30%. Thus, downregulation of FABP5 by curcumin reduces delivery of ATRA to PPARβ/δ, hence reducing RA-mediated transcriptional activation of PPARβ/δ target gene, VEGF-A.

Curcumin regulates multiple transcription factors, including NF-κB. TNBC cells constitutively express NF-κB, a transcription factor that regulates genes known to be involved in proliferation, metastasis and angiogenesis [63]. Having shown previously that NF-κB regulates FABP5 [56], we demonstrate that the mechanism by which curcumin regulates FABP5 gene expression is mediated through the suppression of NF-κB. Hence, FABP5 is regulated by NF-κB in mammary carcinoma MDA-MB-231 cells and curcumin downregulates FABP5 expression by suppressing the p65 subunit of NF-κB.

Limited therapeutic options and poor prognosis of TNBC patients after the treatment with standard conventional drugs creates an emerging need to understand the molecular basis of this disease, as well as to identify alternative chemotherapeutic targets and treatments. A correlation with RA resistance in breast cancer tumors has been attributed to the high expression level of FABP5 [41, 42, 52] which has been associated with poor prognosis in cancer [51]. The fact that TNBC cell lines, MDA-MB-231 and MDA-MB-468, express high levels of FABP5 and are resistant to retinoid therapy suggests that by suppressing the expression level of FABP5, these subtypes of breast cancer cells can overcome RA resistance. Interestingly, our data show that downregulation of the FABP5/PPARβ/δ pathway by curcumin restores the sensitivity of RA-resistant TNBC cells to the inhibitory effects of RA and suppresses cell growth in the combination of curcumin and ATRA. Although a dose of 30 μM curcumin is required to reduce cell growth of MDA-MB-231 cells, it is at this dose that curcumin suppresses the FABP5/PPARβ/δ pathway. Hence, combining ATRA with 30 μM curcumin reduces proliferation an additional 20% and chemosensitizes TNBCs to retinoid therapy. A marginal decrease in cell proliferation by the combination of curcumin and retinoic acid is consistent with the modest suppression of the FABP5/PPARβ/δ pathway. As shown in a previous study, that despite a modest reduction in breast cancer cell proliferation, the combination of metformin and hyperthermia translated to a significant reduction in colony sphere formation [73]. Similarly, synthetic analogues of curcumin, CDF reversed the resistance of pancreatic cells to gemcitabine [74]. Although a 10-20% reduction in cell proliferation was observed in the combination of the two drugs in comparison to CDF alone, the growth inhibitory effects of the combined drugs resulted in significant reduction in colony formation. Despite the modest reduction in our study on mammary carcinoma cell growth with the combination of curcumin and ATRA, this study may translate to a more significant effect *in vivo*. Future studies will explore the effect of curcumin and retinoic acid on the transformational properties of cancer cells to provide information for its use in *in vivo* studies.

Breast cancer cells respond to curcumin at 1–50 μM range with the strongest effect between 20–30 μM [15] . Consistent with our data, several reports have documented that 30 μM curcumin suppresses MDA-MB-231 mammary carcinoma cell growth within the time frame of 48 hours by approximately 40-50% [59, 75–77]. Curcumin has also been studied in several cancer models such as colorectal carcinoma, non small cell lung cancer and pancreatic cancer, and depending on the cancer model, the IC_50_ of curcumin has not only varied among the different cancers, but also between subtypes within a cancer model [78]. One of the criteria that determines the degree to which curcumin can suppress cell proliferation is dependent on the uptake of curcumin within the cells. For instance, MDA-MB-231 cells were more sensitive to the anti-proliferative activity of 25–50 μM curcumin compared to MCF-7 cells [79]. The cellular uptake of curcuminoids in breast cancer cells correlated with the inhibitory activity of this compound which is a determinant of the IC_50_ of curcuminoids in the cancer subtype [79]. Despite the differences in the IC_50_ among cancer cells, one of the advantages of curcumin is its preferential uptake by tumor cells compared to normal cells [80]. Among the strategies used to improve the efficacy of curcumin and potentiate the growth-inhibitory activity of this agent, and more importantly reversing chemoresistance in cancer models, has been aimed at designing and synthesizing novel structural analogues of curcumin [81]. One such compound is demethoxycurcumin which has the potential of suppressing a wide range of mammary carcinoma cell lines with the most efficient inhibitory activity on TNBC (MDA-MB-231 and BT-20) [82]. Analogues of curcumin, such as HO-3867 are taken up by multiple drug resistant or sensitive cancer cell lines at considerably lower doses than curcumin [83], while synthetic analogues, G0-Y030, FLLL-11 and FLLL-12 promotes anti-proliferation in colorectal cancer cells at lower IC_50_ than curcumin [81]. The safety and tolerance of curcumin has been demonstrated in several clinical trials [84], however the disadvantage of curcumin is its poor absorption properties [22]. One such study has shown the improvement in the reduction of cell growth and colony formation of colon cancer stem cells upon treatment with curcumin analog, CDF with 5-fluorouracil and oxaliplatin compared to 5-FU and oxaliplatin with free curcumin [85]. To prevent the emergence on the chemoresistance of cancer cells to conventional chemotherapeutic agents, several studies have employed the use of curcumin to sensitize cancer cells to chemotherapeutic drugs, such as doxorubicin [86]. By improving the absorption of curcumin and cellular uptake of curcumin, curcumin derivatives may be more potent in suppressing the FABP5/PPARβ/δ pathway, and enhance the efficacy of RA by reducing the dosage to be used *in vivo*, in order to better tolerate its use in cancer patients.

Mounting evidence has demonstrated that the expression level of FABP5 is low in low grade tumors and highly upregulated as the progression of the disease manifests to aggressive, metastatic tumors [51, 52, 87–89], clarifying the importance of a rational drug design targeting FABP5. Identifying potential drug targets against FABP5 will establish the sensitivity of cancer cells to RA and may prove to be a rationale to improve the clinical outcome of RA use in breast cancer patients. Our studies demonstrate that curcumin can be used to overcome RA resistance in mammary carcinoma cells since curcumin suppresses FABP5 expression level, reducing the delivery of RA to PPARβ/δ and downregulating the expression of PPARβ/δ target gene. To overcome the challenges of using free curcumin, novel structural analogs of curcumin have been synthesized to improve the chemotherapeutic effects [22, 23]. In addition, curcumin encapsulated in liposomes or production of curcumin nanoparticles has been formulated to enhance the selective delivery of these drugs [90–92]. Using various derivatives of curcumin would improve bioavailability which may improve treatment of breast cancer with RA.

Taken together, we have shown that curcumin in combination with RA sensitizes RA-resistant TNBC cells by suppressing FABP5/PPARβ/δ pathway, and promotes the growth inhibitory effect of RA. Knowing that the acquired resistance to RA in TNBC cells is manifested by the increased expression of FABP5, future studies will entail the development of inhibitors against FABP5. Moreover, we provide evidence that curcumin can inhibit FABP5 and the use of curcumin or its analogs may serve as potential therapeutic agents to overcome RA resistance in RA-resistant breast cancer cells.

## Conclusions

The results of this study showed that curcumin sensitizes RA-resistant TNBC cells to RA-mediated growth suppression. By suppressing the expression level of FABP5 and its cognate receptor, PPARβ/δ, curcumin suppresses the downstream target gene of PPARβ/δ, VEGF-A. The inhibitory effects of curcumin on the FABP5/PPARβ/δ pathway reduce RA-induced VEGF-A expression. The mechanism by which curcumin regulates FABP5 is by downregulating the transcription factor, NF-κB. Further investigation using analogs of curcumin or liposome based curcumin should be employed to evaluate the use of this drug to further enhance efficacy of RA and promote RA sensitivity in RA-resistant breast cancers.

## Abbreviations

FABP5: Fatty acid-binding protein 5
TNBC: Triple negative breast cancer cell
NF-κB: Nuclear factor-kappa B
RA: Retinoic acid
RAR: Retinoic acid receptor
ATRA: All-*trans*-retinoic acid
RARE: Retinoic acid response element
PPARβ/δ: Peroxisome proliferator-activated receptor β/δ
VEGF-A: Vascular endothelial growth factor A
DMSO: Dimethyl sulfoxide
MTT: 3-(4, 5-dimethylthiazol-2-yl)-2, 5-diphenyltetrazolium bromide
qRT-PCR: Quantitative Real-Time Polymerase Chain Reaction
PDTC: Pyrrolidine dithiocarbamate
BrdU: 5-bromo-2’-deoxyuridine.
